# Draft Genome Sequences of Phenotypically Distinct *Janthinobacterium* sp. Isolates Cultured from the Hudson Valley Watershed

**DOI:** 10.1128/genomeA.01426-17

**Published:** 2018-01-18

**Authors:** Alexandra M. Bettina, Georgia Doing, Kelsey O’Brien, Gabriel G. Perron, Brooke A. Jude

**Affiliations:** a Reem-Kayden Center for Science and Computation, Program of Biology, Bard College, Annandale-on-Hudson, New York, USA; b Department of Microbiology and Immunology, Geisel School of Medicine at Dartmouth, Hanover, New Hampshire, USA; c Pediatric Infectious Disease Division, Children’s Hospital of Philadelphia, Philadelphia, Pennsylvania, USA

## Abstract

Investigation of the Hudson Valley watershed reveals many violacein-producing bacteria. These are of interest for their biotherapeutic potential in treating chytrid infections of amphibians. The draft whole-genome sequences for seven *Janthinobacterium* isolates with a variety of phenotypes are provided in this study.

## GENOME ANNOUNCEMENT

The Hudson Valley watershed is home to a large number of vibrantly colored bacterial isolates (1). Many of these Gram-negative *Bacillus* species produce violacein. Production is mediated via a five-gene operon, *vioA–E* (2). Violacein production was first described in *Chromobacterium violaceum* in 1927 (3), and violacein has been widely investigated for its potential biotherapeutic properties; studies have demonstrated bacterial killing (4), fungal killing (5, 6), antiviral activity (7), tumoral cytotoxicity (8, 9), and antinematodal action (10).

Strains BJB1, BJB301, BJB303, BJB304, BJB312, BJB426, and BJB446 were obtained by plating Hudson Valley freshwater sources on R2A agar and incubating at 22 to 25°C for 48 h. All strains initially presented as violet-pigmented colonies and were cultured on R2A, LB, and 1% tryptone agar media. All strains have been characterized for phenotypic behaviors related to growth, motility, quorum sensing, biofilm production, and violacein expression (our unpublished data). Density-dependent phenotypes, including biofilm production and violacein expression, may provide the microorganism with the ability to persist and thrive in a freshwater environment (11).

Genomic DNA extraction was completed with the Qiagen Gentra Puregene Yeast/Bact. kit according to the manufacturer’s protocol. Paired-end Illumina libraries (150 bp) were prepared and HiSeq sequencing was completed using the Illumina HiSeq 4000 instrument (Wright Labs, Huntington, PA). Reads were assembled with a modified version of a previously published local pipeline (12). Adapters and contaminants were scanned for and removed when present. Reads were subsequently quality filtered using BBDuk from the BBMap package version 37.50, keeping to a *Q* score cutoff of 10 (https://sourceforge.net/projects/bbmap). A draft whole-genome assembly was built using SPAdes version 3.11.0 (13) using k-mer sizes of 21, 33, 55, 77, 99, and 127. Contigs shorter than 500 bp or those composed of fewer than four reads were subsequently filtered out of the assembly.

The draft whole-genome assemblies ranged from a high of 70 contigs for BJB426 to a low of 22 contigs for BJB446 (Table 1). The average *N*_50_ value for all seven optimal assemblies was 940,382 bp, with two genomes, those of BJB301 and BJB446, resulting in high *N*_50_ values of 1,349,355 bp and 3,551,037 bp, respectively (Table 1). The average genome size is predicted to be 6.36 Mbp in length, with an average G+C content of 62.85%, comparable to those of published *Janthinobacterium* species (Table 1) (14, 15).

**TABLE 1:** Characteristics of the isolates in this study

Isolate	Origin	Location	No. of contigs	Genome size (bp)	G+C content (%)	*N*_50_ (bp)	Median read depth (×)	Avg no. of CDSs	GenBank accession no.
BJB1	Water	Esopus, Hudson Valley, NY	56	6,353,752	63.35	369,198	730	5,718	PDZO00000000
BJB301	Water	Fishkill Creek, Hudson Valley, NY	24	6,386,165	62.78	1,349,355	504	5,665	PDZJ00000000
BJB303	Water	North Tivoli Bay, Red Hook, NY	48	6,275,771	63.36	319,682	577	5,680	PDZI00000000
BJB304	Water	Norrie Pond, Hudson Valley, NY	62	6,466,089	62.72	218,724	67	5,773	PDZN00000000
BJB312	Water	Dutchess County, NY (vernal pool)	38	6,224,897	62.61	488,122	864	5,559	PDZM00000000
BJB426	Water	Staatsburg, NY	70	6,413,115	62.47	286,558	156	5,711	PDZL00000000
BJB446	Water	Echo Valley, Hudson Valley, NY	22	6,403,346	62.67	3,551,037	316	5,701	PDZK00000000

The assembled contigs were annotated using three methods, a local pipeline running the Prokka genome annotation software (16), the RASTtk annotation software, via the PATRIC pipeline (17, 18), and the NCBI Prokaryotic Genome Annotation Pipeline (PGAP) (19). The 16S rRNA BLAST results for all seven strains aligned most closely with other *Janthinobacterium* species. Annotations across platforms yielded an average of 5,686 coding sequences (CDSs). As expected, a violacein biosynthesis operon (*vioA–E*) was present in all strains. Additionally, all genomes contained genes involved in the bacterial quorum sensing cascade, *jqsA*, *qseC,* and *qseS* (10, 15), as well as twitching motility (*pilT, pilJ, pilH,* and* pilG*), correlating with the phenotypes observed on media.

Further investigation of this large population of related strains may lead to insights into the therapeutic potential of violacein-producing strains.

### Accession number(s).

The whole-genome assemblies have been deposited at DDBJ/ENA/GenBank under the accession numbers listed in Table 1.
